# Mechanisms and Clinical Management of Ventricular Arrhythmias following Blunt Chest Trauma

**DOI:** 10.1155/2016/7270247

**Published:** 2016-02-11

**Authors:** Daniel H. Wolbrom, Aleef Rahman, Cory M. Tschabrunn

**Affiliations:** ^1^St. George's University School of Medicine, St. George's, Grenada; ^2^Department of Surgery, Elmhurst Hospital Center, Icahn School of Medicine at Mount Sinai, New York, NY 11373, USA; ^3^Harvard-Thorndike Electrophysiology Institute, Cardiovascular Division, Department of Medicine, Beth Israel Deaconess Medical Center, Harvard Medical School, Boston, MA 02215, USA

## Abstract

Nonpenetrating, blunt chest trauma is a serious medical condition with varied clinical presentations and implications. This can be the result of a dense projectile during competitive and recreational sports but may also include other etiologies such as motor vehicle accidents or traumatic falls. In this setting, the manifestation of ventricular arrhythmias has been observed both acutely and chronically. This is based on two entirely separate mechanisms and etiologies requiring different treatments. Ventricular fibrillation can occur immediately after chest wall injury (commotio cordis) and requires rapid defibrillation. Monomorphic ventricular tachycardia can develop in the chronic stage due to underlying structural heart disease long after blunt chest injury. The associated arrhythmogenic tissue may be complex and provides the necessary substrate to form a reentrant VT circuit. Ventricular tachycardia in the absence of overt structural heart disease appears to be focal in nature with rapid termination during ablation. Regardless of the VT mechanism, patients with recurrent episodes, despite antiarrhythmic medication in the chronic stage following blunt chest injury, are likely to require ablation to achieve VT control. This review article will describe the mechanisms, pathophysiology, and treatment of ventricular arrhythmias that occur in both the acute and chronic stages following blunt chest trauma.

## 1. Introduction

Under certain conditions, sustained ventricular arrhythmias can develop immediately or late in the setting of nonpenetrating, blunt chest trauma. The manifestation of ventricular arrhythmias in this setting is a rare, but potentially fatal complication that can include triggered, automatic, and reentrant ventricular arrhythmias. The acute manifestation of ventricular fibrillation (VF) in this setting, often referred to as commotio cordis, has been well described and occurs in the absence of cardiac structural injury [1]. The development of monomorphic ventricular tachycardia (VT) and/or ventricular premature depolarizations (VPDs) beyond the acute phase following blunt chest trauma is more commonly associated with right or left ventricular structural abnormalities but with little understanding of the underlying mechanisms or recommended therapies. We will discuss both of these phenomena in this review along with current treatment and available outcome data.

## 2. Review of Ventricular Arrhythmia Mechanisms in Blunt Chest Trauma

### 2.1. Commotio Cordis, Ventricular Fibrillation

Since the mid-1800s, nonpenetrating, blunt chest trauma, with no structural cardiac damage, resulting in sudden cardiac death has been described in the literature using the Latin term,* commotio cordis*. Although case studies have been published describing isolated incidents since the 19th century, it was not until the establishment of the United States Commotio Cordis Registry in 1996 that we began to understand the significance of this phenomenon. Since the inception of the registry, about 10 to 20 cases are reported annually to the database and have allowed for a better understanding of the prevalence and public health implications necessary to increase survival rates [2, 3].

**Figure 1:**
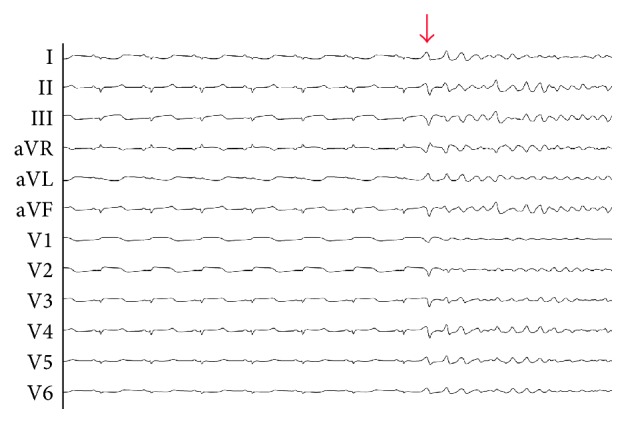
Ventricular fibrillation initiated by premature ventricular depolarization.

Commotio cordis is defined as the “mechanical stimulation of the heart by non-penetrating, impulse-like impact to the precordium that, through intrinsic cardiac mechanisms, gives rise to disturbances of cardiac rhythm of varying type, duration, and severity, including sudden cardiac death, in the absence of structural damage that would explain any observed effects” [4]. In this condition, mechanical stimulation of the heart by nonpenetrating impact to the precordium gives rise to VF (Figure 1). Since the establishment of the United States Commotio Cordis Registry, more than 220 cases have been reported [1]. From this database, we can see that 75% of these cases took place during either competitive or recreational sporting event. Typically, the patient is a young male competing or participating in a sporting activity who is struck with a small, dense projectile like a baseball or hockey puck at a high velocity over the cardiac silhouette [5–7]. It is not known why young males experience this rare phenomenon more often, but it is suspected that younger individuals have higher chest compliance compared with older adults, and males, who typically play more compact sports, are important risk factors for this condition [1].

This is a rare phenomenon due to the required synchronization of certain pathophysiological variables. During impact-related animal model studies, commotio cordis inducing VF can be prompted with impacts at a certain critical time of the cardiac cycle. Specifically, this time period includes the upslope of the T-wave, about 10 to 40 milliseconds prior to the T-wave peak. Typically, as a result of this impact, the morphology appears initially uneven and undulating, described as either a polymorphic ventricular tachycardia (PVT) or Torsade de Pointes. While PVTs are typically observed at first, they will progress into VF if sustained for over 10 beats. Outside this time frame, VF was rarely seen. In addition to the narrow time period where the cardiac rhythm is susceptible to electrical abnormalities secondary to a direct blow over the cardiac silhouette, successively higher speeds of impact increased the likelihood of VF in swine models up until 80 km/h (50 mph). Between 80 and 112 km/h (50–70 mph), the likelihood of VF dropped and increased the likelihood of cardiac rupture. Other determinants that play a key role in VF production include location, energy, shape, and hardness of the object [1, 8].

The mechanisms in in vivo studies indicate that a high speed impact with a dense projectile to the chest causes premature ventricular depolarization, coupled with elevated ventricular stretch-related activation of mechanosensitive ion channels such as ATP-dependent K^+^ channels. This mechanism can result in the ventricular arrhythmias described above and, occasionally, will result in sudden cardiac death. Additional investigations of the characteristics of mechanical stretch in commotio cordis indicate that Ca^+2^ waves are initiated by damaged myocardial tissue, whereby nonuniform muscles are stretched during contraction triggering VF [9, 10]. Furthermore, several additional individual variables have been noted to increase VF incidence in multiple animal studies and patient case reports [11, 12]. In general, variations of wider QRS and longer QT periods at baseline (possibly influenced by genetic differences in genotypes of repolarization channels) have higher susceptibility to VF.

### 2.2. Monomorphic Ventricular Tachycardia due to Electrical and Structural Ventricular Remodeling Long after Blunt Chest Injury

**Figure 2:**
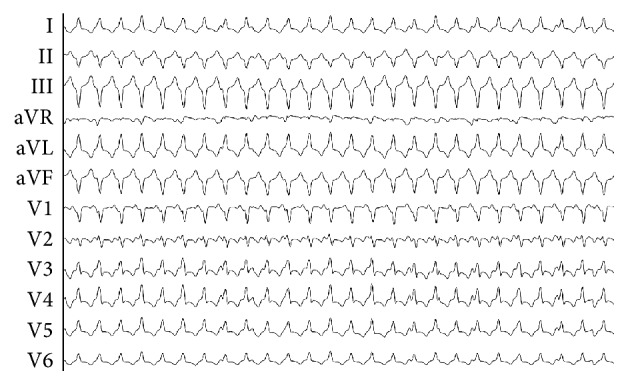
Reentrant ventricular tachycardia.

A second manifestation of ventricular arrhythmias following blunt chest trauma is the development of ventricular tachycardia (Figure 2) due to ventricular aneurysm and/or tissue fibrosis that develops long after the initial injury. Very rarely, blunt chest injury leads to electrical and structural ventricular remodeling that can serve as the underlying substrate for the development of sustained monomorphic VT well beyond the acute setting [13, 14]. This phenomenon is not well described and is poorly understood as opposed to commotio cordis, and a review of the literature has revealed no long-term epidemiological studies of this specific phenomenon.

In this setting, the traumatic event results in permanent structural damage to the heart muscle most commonly involving the right ventricle (RV) and may result in RV aneurysmal development. The RV is more prone to cardiac contusion and subsequent thinning of the ventricular wall because of its anterior location within the chest. While traumatic RV aneurysms are uncommon, these rare cases usually present outside the acute setting of the injury, generally found incidentally during autopsy, or following the manifestation of clinical symptoms of ventricular tachycardia (i.e., palpitations, near-syncope, etc.) [13, 15]. We performed a systematic literature review for such cases and only identified 7 instances of chronic sustained VT development from a remote blunt chest wall injury. Nonetheless, it is important to note that RV aneurysm development rarely results in the development of ventricular tachycardia [13, 14, 16]. Patients that do develop abnormal ventricular substrate leading to VT beyond the acute injury phase appear to require an aggressive management and treatment approach. Of note, 2/7 of these patients did not have evidence of any overt cardiac structural changes and the electrophysiology studies were highly suggestive of a focal mechanism in both cases.

The other 5 patients did have evidence of an underlying abnormal structural pathophysiology including aneurysm and/or scar development that was involved in the clinical VT. This is highly suggestive of a causal relationship of chest wall injury to the development of cardiac structural changes and most likely a reentrant VT likely due to inhomogeneous scarring with a variable degree of surviving myocardial tissue. The resulting arrhythmogenic substrate is characterized by zones of slow conduction due to nonuniform anisotropy, resulting in fixed and/or functional regions of conduction block. This facilitates reentry as it generates enough time for tissue in the circuit to recover its excitability to allow the excitation wave front to reenter the initial site of the block, thereby creating a reentrant circuit [17, 18].

## 3. Implications and Clinical Management

### 3.1. Immediate and Acute Ventricular Arrhythmia Manifestation

In a study by Link et al., juvenile swine were used to demonstrate the effectiveness of defibrillation for ventricular arrhythmias as a result of nonpenetrating, blunt chest trauma in the absence of structural injury. In the 2003 study, swine were analyzed with automated external defibrillators (AEDs) 30 seconds after receiving a blow to the chest by a baseball at 64 km/h (40 mph), inducing VF. The animals were defibrillated with a 200 J biphasic waveform, escalated to 300 J and then 360 J if needed, and were randomly assigned to groups and defibrillated at either 1-, 2-, 4-, or 6-minute intervals without prior cardiopulmonary resuscitation (CPR). The study demonstrated that the AED device correctly analyzed the VF in the 50 baseball induced ventricular arrhythmias in all cases and was able to successfully terminate the ventricular rhythm with single 200 J defibrillation in 94% cases. The other three events required additional defibrillation to terminate the arrhythmia but were ultimately successful with 3 or less shocks 100% of the time [8, 23].

The time from the traumatic event to initial shock was a significant predictor in the survival of the swine, according to the 2003 study by Link et al. The swine defibrillated at a one-minute interval had a 100% (13 of 13) survival rate; the swine defibrillated at 2 minutes had a 92% survival rate (11 of 12); survival at 4 minutes was 46% (6 of 13); and, finally, initial defibrillation at 6 minutes had a survival rate of 25% (3 of 12). This clearly demonstrates the importance in early recognition and defibrillation as critical factors to ensure effective management of commotio cordis patients.

Although the animal studies have shown the effectiveness of early defibrillation for acute ventricular arrhythmias secondary to blunt chest trauma, data over the last 15 to 20 years has demonstrated that the outcomes are generally poor. The survival rate from 1996 to 2014 is approximately 25% [1]. However, as public awareness of commotio cordis and AED access has increased over the past few years, the survival rate has begun to increase. A study analyzing the survival rates from 2006 to 2012 demonstrated that the survival rate has more than doubled to 58% (31 of 53 events) [3].

The American Heart Association has recommended through various public awareness campaigns and training courses that any sudden collapse of an athlete after a blow to the chest from a solid projectile should be considered commotio cordis with rapid initiation of CPR and defibrillation. Immediate recognition is key to ensuring early resuscitation is initiated and remains a significant factor influencing survival from commotio cordis. Increased awareness has led to the wide distribution of AEDs in both public places, like parks, as well as many sporting venues [24]. These widely distributed AEDs, which are now available for laypersons to use when they recognize commotio cordis, can be used immediately after the onset of the ventricular arrhythmias. The combination of recognizing commotio cordis and initiating resuscitative efforts and the wide availability of AEDs are the most significant changes in the past few years which have likely led to the increased survival rates [5].

### 3.2. Chronic Ventricular Arrhythmia Manifestation

The management of chronic ventricular arrhythmias is less clear due to its infrequent nature and associated structural changes. A comprehensive literature review suggests that the incidence of ventricular arrhythmias due to ventricular structural changes is exceptionally rare. We identified only 7 such cases published between 1998 and 2014 and have outlined the clinical presentation, treatment, and follow-up in Table 1 and in further detail below. All patients presented with palpitations and recurrent episodes of monomorphic VT. Four patients (67%) presented with a left bundle branch block VT morphology consistent with a right ventricular origin and 3 patients had right bundle branch block morphologies suggestive of LV origin. Three of the 4 patients with RV VT had structural abnormalities identified during noninvasive imaging in the form of RV free wall thinning and/or aneurysm. One patient with LV VT did not have overt structural changes identified and the other LV VT patient had evidence of LV epicardial lateral wall contact with a previous fractured rib that had experienced blunt chest injury 2 years priorly. Three cases had documented ventricular arrhythmias acutely following the initial chest injury which continued during long-term follow-up. The other 3 cases manifested symptoms and monomorphic VT for the first time at variable time points and ranged from 6 months to 20 years after the initial chest wall injury. Importantly, 6/7 (86%) of patients continued to have VT despite pharmacologic and antiarrhythmic drug therapy requiring electrophysiology study, cardiac mapping, and ablation therapy. Epicardial radiofrequency ablation or surgical cryoablation was performed in 3 cases and 1 patient underwent aneurysm repair. All 6 case reports undergoing ablative/surgical treatment indicated success at eliminating the patients' arrhythmia without recurrence during an average follow-up of 11.8 ± 7.2 months (range: 6–24 months) off antiarrhythmic medications. Only 1 patient in this series with a left bundle, superior axis VT and inferolateral RV thinning on CMR was implanted with an internal cardioverter-defibrillator (ICD) and did not undergo ablation as there was only NSVT and there were no detected ICD events during 22-month follow-up.

*Case 1*. Mera et al. report their experience in a 36-year-old previously healthy man that presented following a freight train collision that involved blunt chest wall injury [19]. The patient complained of palpitations associated with frequent monomorphic VPDs identified on telemetry. Several hours later while in the hospital, he developed symptomatic left bundle, inferior axis monomorphic VT which self-terminated after the patient had syncopized. Transthoracic echocardiogram and coronary angiography were normal. The patient continued to complain of palpitations and dizziness several months later with frequent VPDs and nonsustained VT despite *β*-blockers and sotalol. Subsequent electrophysiology study localized the VPD site of origin to the RVOT. Radiofrequency ablation at this site successfully eliminated the ectopic beats and remained asymptomatic during a 2-year follow-up. Although the ventricular arrhythmias appear to have manifested immediately after chest wall injury and continued during follow-up, the focal mechanism of the arrhythmia, absence of significant cardiac contusion, and normal cardiac function on echocardiogram suggest that this patient may have had idiopathic RVOT VPDs/VT unrelated or further elucidated after the blunt chest injury. 

*Case 2*. Martínez et al. describe the case of a 24-year-old female with ongoing palpitations and sustained right bundle branch block VT at 205 bpm suspected to involve the LV apical region [20]. The patient had suffered a severe blunt chest injury involving a rock 20 years priorly. She did not develop any acute complications after this event, but ventriculography demonstrated apical aneurysm with normal coronary angiography indicating myocardial contusion and injury eventually resulting in LV apical scar and aneurysm. The patient underwent aneurysm resection with Teflon and pericardial patch insertion. Repeat EP study with RV programmed extrastimuli at three cycle lengths failed to induce any arrhythmias. The patient remained asymptomatic without VT recurrence during 12-month follow-up. Characterization of the VT mechanism was not performed, but the underlying aneurysm and elimination of VT following resection are suggestive of reentry. This case is similar to the more common scenario of patients that develop LV aneurysms and monomorphic VT following myocardial infarction. Subendocardial resection has been shown to be a highly effective treatment for recurrent VT in a majority of post-MI patients [25].

*Case 3*. Schaer et al. describe a patient who is a 33-year-old male hockey player who began experiencing palpations after a body-check during a professional ice hockey game [16]. A left bundle superior axis monomorphic VT was induced but not targeted with ablation therapy. The patient discontinued his professional sports activities but did not receive any further treatment. A CMR performed a year after the injury showed a small area of myocardial thinning in the inferolateral wall of the RV. It was suspected that the body-check that resulted in palpations acutely after the chest trauma was the cause of the subsequent RV structural defect. Nearly 2 years later, the patient reported similar symptoms after another body-check during a recreational ice hockey game. The patient demonstrated VT with the same morphology as the original incident and underwent cardioversion and ICD implantation. A 2-year follow-up revealed no additional episodes of palpations or ICD detected events. 

*Case 4*. Horduna et al. report a pediatric case in a 10-year-old boy referred to VT management 3 years after being struck by a car [21]. The patient developed monomorphic VT shortly after the traumatic injury during prolonged hospitalization. The patient continued to develop sustained VT with a right bundle, superior axis VT despite verapamil and sotalol therapy. CMR and echocardiogram were normal. The electrophysiology study and endocardial left ventricular electroanatomic activation mapping were performed during VT and identified a focal source of early activation at the mid-portion of the anterolateral LV consistent with an anterolateral papillary muscle VT. Radiofrequency ablation was delivered at this site and resulted in VT termination without recurrence after 9 months off all antiarrhythmic medications. Although this patient did not have evidence of mitral valve injury or ventricular scar on CMR imaging and endocardial bipolar voltage mapping, this VT site of origin is highly suggestive of papillary muscle injury that occurred during the initial blunt chest traumatic event. The pathophysiologic result from this injury and its role in the manifestation of VT are unclear. It is possible that tissue changes in this region causing the suspected nonautomatic focal VT mechanism are subtle and beyond the detectable resolution of CMR imaging and bipolar voltage mapping. 

*Case 5*. Michowitz et al. report a case of recurrent VT in a 59-year-old man that had been kicked in the chest by a horse 20 years earlier [14]. No acute arrhythmias or abnormal clinical manifestations were observed initially, but subsequent RV dilation was seen on serial echocardiography. Due to amiodarone toxicity, it was decided that the patient would discontinue use of the antiarrhythmic medication and undergo electrophysiology study and ablation. Imaging prior to the procedure revealed a dilated RV and ECG showed right bundle branch block. Endocardial RV electroanatomic mapping demonstrated free wall and septal bipolar low voltage indicative of tissue fibrosis. Mid-diastolic potentials were seen during VT activation mapping more likely indicative of a reentrant versus focal mechanism. Endocardial ablation was performed but was unsuccessful in terminating the VT, prompting epicardial access, mapping, and ablation. However, epicardial ablation did not terminate the VT. Only after further RV endocardial lesions were delivered was late VT termination seen. Late termination from the RV endocardium after endocardial and epicardial ablation may have been due to a mid-myocardial RV circuit. The patient remained free of any VT recurrence during 6-month follow-up. 

*Case 6*. Casado-Arroyo et al. report a case of recurrent right bundle, superior axis sustained monomorphic VT in a 62-year-old man that had a traumatic blunt chest wall injury from a fall 2 years priorly [22]. The patient developed palpitations, dizziness, and depressed left ventricular function. Computer tomographic (CT) imaging identified LV lateral wall contract with prior rib fractures consistent with the VT electrocardiographic morphology. Epicardial surgical mapping was performed and identified a signal 60 ms pre-QRS at the LV lateral wall. Cryoablation at this site resulted in immediate VT termination and elimination. The presystolic electrogram and the absence of overt structural heart disease, despite the particularly unusual CT finding, are suggestive of a focal VT mechanism. The patient denied any further symptoms and left ventricular function returned to normal during a 12-month follow-up. 

*Case 7*. Shakil et al. report a case of left bundle branch block NSVT morphology in a 33-year-old man who presented with palpations for 2 weeks [13]. The patient experienced a blunt chest injury during a skiing accident 6 months prior to the symptoms and, subsequently, it was hypothesized that the injury was the impetus behind the NSVT and related symptoms. An exercise tolerance test was attempted multiple times but was discontinued each time because of sustained VT that resolved with rest. The patient was admitted to the hospital and underwent a contrast-enhanced CT angiography. The imaging revealed myocardial dilatation in the RV free wall near the annulus of the tricuspid valve consistent with aneurysm and fibrosis. Endocardial RV mapping and ablation of the VT were attempted but were unsuccessful due to anatomic limitations and challenges reaching the inferior RV aneurysm. The patient was initiated on amiodarone and *β*-blocker therapy but ultimately refused long-term antiarrhythmic drug use. The patient elected to undergo a hybrid surgical procedure involving RV epicardial mapping and cryoablation of the RV aneurysm. The patient was discharged 4 days later after an uneventful postoperative stay and remained free of any further VT off antiarrhythmic medications during 8-month follow-up.

In the past, medical management with antiarrhythmic drugs was the standard choice of treatment among clinicians to control ventricular arrhythmias. Today, we know that 40% of patients do not respond to this type of treatment for VT [13, 26]. For patients who do not respond to the antiarrhythmic drugs or have some other contraindications, such as toxemia, other options are available. As we described in the case reports presented in this review, these other options are endocardial resection, repair of the aneurysm, and radiofrequency/cryoablation [27].

## 4. Conclusions

Nonpenetrating, blunt chest trauma is a serious medical condition with multiple types of clinical presentations and variable prognoses. Typically, blunt chest trauma is a result of dense projectiles used during competitive and recreational sports, but various other etiologies have been described, including body-checks during ice hockey, horse kicks to the chest, and ski related injuries. Ventricular arrhythmias can develop acutely or long after the event, each occurring based on two entirely separate pathophysiologies with different treatment requirements. Ventricular fibrillation can occur immediately after chest wall injury (commotio cordis) and requires rapid defibrillation. Reentrant ventricular tachycardia can develop in the chronic stage due to underlying structural heart disease long after blunt chest injury. The associated arrhythmogenic tissue may be complex and provides the necessary substrate to form a reentrant VT circuit. Ventricular tachycardia in the absence of overt structural heart disease appears to be focal in nature with rapid termination during ablation. Regardless of the VT mechanism, patients with recurrent episodes despite antiarrhythmic medication in the chronic stage following blunt chest injury are likely to require ablation to achieve VT control. This was associated with a high degree of success during clinical follow-up off antiarrhythmic drugs.

## Figures and Tables

**Table 1:** Ventricular tachycardia long after BCI case findings.

Case	Age/gender	Symptoms	Time from BCI to Tx	SHD	VT morph and SOO	Tx	Success	Follow-up
(1) Mera et al., 1998 [19]	36 M	Palpitations Syncope	“Several months”	N	LB, inferior axis RVOT	CA	Y	24 months

(2) Martínez et al., 2003 [20]	24 F	Palpitations	20 years	Y	RBBB 205 bpm LV apex	Aneurysm resection	Y	12 months

(3) Schaer et al., 2007 [16]	33 M	Palpitations	22 months	Y	LB, superior axis 300 bpm RV free wall	ICD	—	24 months NSVT only

(4) Horduna et al., 2011 [21]	10 M	Palpitations	3 years	N	RB, superior axis 110 bpm LV anterolateral PM	CA	Y	9 months

(5) Michowitz et al., 2012 [14]	59 M	ICD therapy	20 years	Y	LB, inferior axis 130 bpm RV free wall	CA	Y	6 months

(6) Casado-Arroyo et al., 2012 [22]	62 M	Palpitations Dizziness	2 years	Y	RB, superior axis 160 bpm LV lateral wall	SA	Y	12 months

(7) Shakil et al., 2012 [13]	33 M	Palpitations	6 months	Y	LBBB RV free wall	SA	Y	8 months

M, male; F, female; BCI, blunt chest injury; VT, ventricular tachycardia; Tx, treatment; SOO, site of origin; CA, catheter ablation; SA, surgical ablation; NSVT, nonsustained ventricular tachycardia; LB, left bundle; RB, right bundle; ICD, internal cardioverter-defibrillator.
